# Milk Fermented by Specific *Lactobacillus* Strains Regulates the Serum Levels of IL-6, TNF-α and IL-10 Cytokines in a LPS-Stimulated Murine Model

**DOI:** 10.3390/nu10060691

**Published:** 2018-05-29

**Authors:** Aline Reyes-Díaz, Verónica Mata-Haro, Jesús Hernández, Aarón F. González-Córdova, Adrián Hernández-Mendoza, Ricardo Reyes-Díaz, María J. Torres-Llanez, Lilia M. Beltrán-Barrientos, Belinda Vallejo-Cordoba

**Affiliations:** Centro de Investigación en Alimentación y Desarrollo, A.C. (CIAD), Carretera a La Victoria Km. 0.6, Apartado 1735, Hermosillo, Sonora 83304, Mexico; alredi22@hotmail.com (A.R.-D.); vmata@ciad.mx (V.M.-H.); jhdez@ciad.mx (J.H.); aaronglz@ciad.mx (A.F.G.-C.); ahernandez@ciad.mx (A.H.-M.); ricardo.reyes@ciad.mx (R.R.-D.); mtorres@ciad.mx (M.J.T.-L.); lilia.beltranb@gmail.com (L.M.B.-B.)

**Keywords:** fermented milk, cytokine regulation, *Lactobacillus*, lipopolysaccharide

## Abstract

Studies report that metabolites, such as peptides, present in fermented milk with specific lactic acid bacteria, may regulate cytokine production and exert an anti-inflammatory effect. Hence, the cytokine regulatory effect of fermented milk by specific *Lactobacillus* strains was evaluated in a lipopolysaccharide (LPS)-stimulated murine model. From twelve strains, three (J20, J23 and J28) were selected for their high proteolytic and acidifying capacities in milk and used for the in vivo study. Three treatments (fermented milk, FM; pasteurized fermented milk, PFM; and its <10 kDa fractions, PFM10) were administrated daily for four weeks. After treatments, animals were induced to a systemic inflammation with LPS, and blood samples were collected 6 h post-LPS injection for cytokine analyses. Results showed that FM or PFM significantly (*p* > 0.05) reduced pro-inflammatory cytokine (IL-6 and TNF-α) concentrations and significantly increased anti-inflammatory (IL-10) cytokine concentrations in comparison to the control; also, pro-inflammatory cytokines were reduced for animals treated with PFM10 (*p* < 0.05). RP-HPLC-MS/MS analysis showed that water-soluble extracts (<10 kDa) from PFM with J28 presented 15 new peptides, which may be the metabolites involved in the cytokine regulatory effect of fermented milk.

## 1. Introduction

The intake of dairy products fermented by *Lactobacillus* (*L*.) spp. have been associated with the ability to confer health benefits [1]. The modulation of the specific immune response through cytokine production regulation, which has shown to be strain dependent, is probably the most important health benefit [2,3]. The health-promoting effects may arise not only from bacteria themselves [4], but also from metabolites derived from milk fermentation, particularly bioactive peptides, that play a crucial role on these beneficial effects [5]. However, little attention has been paid to the modulation of cytokine production by bioactive peptides produced in milk fermented by lactic acid bacteria (LAB). 

It has been reported that cell wall components of LAB or peptides liberated in milk may interact with specific receptors in the immune cells, which selectively influence the immune system through different mechanisms [6]. In fact, peptides derived from *L. helveticus*-fermented milk could enhance the systemic immune response following an *Escherichia* (*E.*) *coli* O157:H7 challenge [6]. Nevertheless, few reports have been published on the anti-inflammatory effects of fermented milk with other *Lactobacillus* strains. Indeed, most in vitro and in vivo related studies have been carried out with milk fermented with *L. helveticus* [7,8,9], and only one study was performed with *L. fermentum* [10].

Lipopolysaccharide (LPS) induces acute inflammation and endotoxic shock in experimental animals [3,11,12,13], and it is characterized by the production of cytokines and other mediators of inflammation. Therefore, LPS may be used to induce inflammation to evaluate the anti-inflammatory effect of fermented milk. During an infection by bacteria or viruses, among others, the host responds producing pro-inflammatory cytokines to contain infection. Pro-inflammatory cytokines such as interleukin-6 (IL-6) and tumor necrosis factor alpha (TNF-α) are responsible for early responses and amplify reactions, whereas anti-inflammatory cytokines such as IL-4 and IL-10 reduce inflammation and promote healing [14]. Pro-inflammatory cytokines contribute to defense mechanisms of the host and when secreted in excess, they may induce immunopathological disorders [15,16,17,18]. Therefore, it is necessary to find ways to downregulate its production or inhibit its effects in vivo. The aim of the present study was to evaluate the anti-inflammatory effects of milk fermented by specific *Lactobacillus* strains, on the serum cytokines levels in a LPS-stimulated murine model. Firstly, *Lactobacillus* strains were selected based on their technological properties such as proteolytic and acidifying activities. Then, selected strains were used to prepare fermented milk (FM), pasteurized fermented milk (PFM) and its fractions (<10 kDa) (PFM10), and the anti-inflammatory effects were tested in an in vivo study. In addition, peptides present in fractions (<10 kDa) were identified, since they may be associated to the potential anti-inflammatory effect.

## 2. Materials and Methods

### 2.1. Substrates and Chemicals

Lactobacilli MRS broth (De Man, Rogosa and Sharpe) was purchased from Difco (Sparks, MD, USA). Nonfat dry milk was obtained from Dairy America (Fresno, CA, USA). O-Phthaldialdehyde (OPA) was purchased from Fluka (Linz, Oberösterreich, Austria), and trichloroacetic acid (TCA), sodium dodecyl sulfate (SDS), 2-mercaptoethanol, acetonitrile, trifluoroacetic acid (TFA) and LPS from *E. coli* serotype O11:B4 were purchased from Sigma Chemical Co. (St. Louis, MO, USA).

### 2.2. Strains and Growth Conditions

Twelve *Lactobacillus* strains were obtained from the culture collection from the Dairy Laboratory at the Food Research and Development Center, A.C. (CIAD, A.C., Hermosillo, Sonora, Mexico). These wild strains were isolated from artisanal cheese and characterized [19]. *Lactobacillus* strains were cultured in 10 mL of sterile MRS Broth. Then, three consecutive subcultures were prepared (1%, *v*/*v*) and incubated for 24, 18 and 12 h, respectively, at 37 °C, in order to obtain fresh cultures. The initial average population (10^9^ CFU/mL) of the inoculum was obtained in the 12-h fresh culture by the plate count method.

### 2.3. Preparation of Fermented Milk

Reconstituted nonfat dry milk (10%, *w*/*v*) was heated at 110 °C for 10 min, immediately cooled and stored overnight at 4 °C. To prepare starter cultures, a fresh culture of each strain was inoculated (1%, *w*/*v*) in sterile reconstituted nonfat milk, followed by incubation at 37 °C for 12 h. FM was produced by inoculation of sterile reconstituted commercial nonfat dry milk (10%, *w*/*v*) with starter cultures (3%, *v*/*v*), followed by incubation at 37 °C for 48 h. The final average population in each FM was 109 CFU/mL. PFM was prepared by heating fermented milk at 75 °C for 20 min and immediately cooling in an ice bath. PFM10 was prepared by centrifuging PFM in a Sorvall ST 16R centrifuge at 4696× *g* (Thermo Scientific, Osterode, Am Kalkberg, Germany) for 40 min at 4 °C; and supernatants were collected and ultra-filtered through 10 kDa cut-off membranes (Pall life Science, Port Washington, NY, USA) in a stirred ultrafiltration cell (Millipore Amicon, Bedford, MA, USA). Permeates (<10 kDa) were collected, frozen at −80 °C and lyophilized in a FreeZone 6 freeze dryer (Labconco, Kansas, MO, USA). For the in vivo study, PFM10 was daily prepared by reconstitution to the original volume. Meanwhile, control acidified milk (AM) was prepared with sterile nonfat milk and acidified with lactic acid (Faga Lab, Cd. México, México) until a pH of 4.3 was reached. Peptide content in PFM and PFM10 was determined in the water-soluble fractions after protein precipitation with TCA (14%) by the bicinchoninic acid (BCA) method [20].

### 2.4. Proteolytic and Acidifying Activity

Proteolysis during milk fermentation at 24 and 48 h was quantified by using the OPA method [19]. For this purpose, 5 mL of 0.75 N TCA were added to 2.5 mL of fermented milk and vortexed for 1 min. Samples were kept at 4 °C for 30 min, then they were centrifuged (4696× *g*, 40 min, 4 °C). The supernatants were filtered using Whatman No. 2 paper. All filtrates were frozen at −20 °C until further analysis. A 30 μL sample aliquot containing TCA-soluble peptides were added to 600 μL of the freshly prepared OPA reagent. After 2 min at 20 °C, absorbance was immediately measured at 340 nm using a spectrophotometer Nanodrop 2000C (Thermo Scientific, Claire, WI, USA).

During fermentation, pH measurements were taken at 24 and 48 h, using a HI 2211 pH and ORP Benchtop Meter (Hanna Instruments, Woonocket, RI, USA). Additionally, titratable acidity was monitored [21]. Measurements were taken in duplicate.

### 2.5. Assay in a Murine Model

Male Wistar rats (5–6 weeks old, 110–160 g body weight (BW)) were obtained from Bioinvert & Aprexbio S.A. de C.V. (Ciudad de México, México). All rats were fed with a standard diet (Standard Rodent Laboratory-Chow 5001 Diet, Purina Feeds, Inc., St. Louis, MO, USA) and purified water *ad libitum*. Rats were housed in sanitized polycarbonate cages (60 × 40 × 30 cm) with sterile sawdust bedding that was replaced daily. Room temperature was kept at 22 ± 2 °C, with a relative humidity between 40 and 60%, and 12 h light/dark cycles.

Animals were adapted (first week) and randomly assigned in pairs to experimental groups (*n* = 6). The bioassay included the eleven groups: AM (control), FM/20, PFM/J20, PFM10/J20, FM/J23, PFM/J23, PFM10/J23, FM/J28, PFM/J28, and PFM10/J28. Also, a PBS (phosphate-buffered saline solution) group without LPS-induced inflammation was included for the evaluation of cytokine concentrations in a nonstimulated group. During four weeks, animals were daily fed with 1 mL of each treatment.

After four weeks, animals were subcutaneously injected with LPS (7.5 mg/kg BW, diluted in milliQ water) to induce a systemic inflammatory process. Finally, rats were sacrificed 6 h post-LPS stimulation. Blood samples were taken and centrifuged at 2348× *g* for 8 min; serum was collected and kept at −20 °C until cytokine analyses. This study was approved by the ethical committee of the Food Research and Development Center, A.C. (CE/007/2015) and was carried out following the recommendations of the Committee on Care and Use of Laboratory Animals of the Institute of Laboratory Animals Resources [22].

### 2.6. Cytokine Determinations

IL-10, IL-6 and TNF-α serum cytokine concentrations were determined by the ELISA method (Enzyme-Linked Immunosorbent Assay) using commercially available kits (Thermo Scientific, Rockford, IL, USA). These tests comprised recombinant cytokines from *E. coli* and antibodies against anti-inflammatory and pro-inflammatory cytokines (with 3, 5 and 15 pg/mL detection limits, respectively).

### 2.7. Isolation of Peptide Fractions by Reversed-Phase HPLC

Peptide profiles of PFM10 samples were obtained by reversed-phase high performance liquid chromatography (RP-HPLC) (1100 series; Agilent Technologies Japan Ltd., Tokyo, Japan) at 214 nm. Separation was carried out with an Extend-C18 (4.6 × 250 mm, 5-μm particle size, 180-A pore size) column from Agilent Technologies (Santa Clara, CA, USA) at room temperature (22 °C) with a solvent flow rate of 0.25 mL/min. Solvent A was a mixture of water-trifluoroacetic acid (1000:0.4, *v*/*v*) and solvent B contained acetonitrile-trifluoroacetic acid (1000:0.3, *v*/*v*). 20 µL of sample were injected. Peptides were eluted with a linear gradient of solvent B in solvent A from 0.1 to 99.9% for 40 min. The concentration of solvent B was linearly increased from 0.1 to 60 % in 30 min and from 60 to 99.9% between 30 and 35 min, then it was decreased from 99.9% to 0.1% between 35–40 min. Fractions from five chromatographic runs were collected, freeze dried and stored for further analysis.

### 2.8. Analysis of Peptides by Tandem Mass Spectrometry

Mass spectrometry (MS) analysis was performed using a 1100 Series LC/MSD Trap (Agilent Technologies Inc., Waldbronn, Karlsruhe, Germany) equipped with an electrospray ionization source (LC-ESI-MS). The nanocolumn was a C18-300 (150 mm × 0.75 µm, 3.5 µm; Agilent Technologies Inc.). The sample injection volume was 1 µL. Solvent A was a mixture of water-acetonitrile-formic acid (10:90:0.1, *v/v/v*) and solvent B contained water-acetonitrile-formic acid (97:3:0.1, *v/v/v*). The gradient was based on the increment of solvent B, which was initially set at 3% for 10 min and it took 23 more min to reach 65%. The 0.7 µL/min flow rate was directed into the mass spectrometer via an electrospray interface. Nitrogen (99.99%) was used as the nebulizing and drying gas and operated with an estimated helium pressure of 5 × 10^2^ Pa. The needle voltage was set at 4 kV. Mass spectra were acquired over a range of 300 to 2500 mass/charge (*m*/*z*). The signal threshold to perform auto MS analyses was 30,000. The precursor ions were isolated within a range of 4.0 *m*/*z* and fragmented with a voltage ramp from 0.35 to 1.1 V. Peptide sequences were obtained from mass spectrometry data using the Mascot [23] server through the UniProtKB/Swiss-Prot database (http://www.matrixscience.com/help/seq_db_setup_Sprot.html (accessed on 5 September 2016) sequences.

### 2.9. Statistical Analysis

Data normality was tested as a prerequisite before one way analysis of variance (ANOVA) was carried out in order to compare groups. Differences among means were assessed by Fisher’s least significant difference for multiple comparison test and considered significant when *p* ≤ 0.05. Data analyses were performed with NCSS 2007.

## 3. Results and Discussion

### 3.1. Strain Selection

#### 3.1.1. Proteolytic Activity

**Figure 1:**
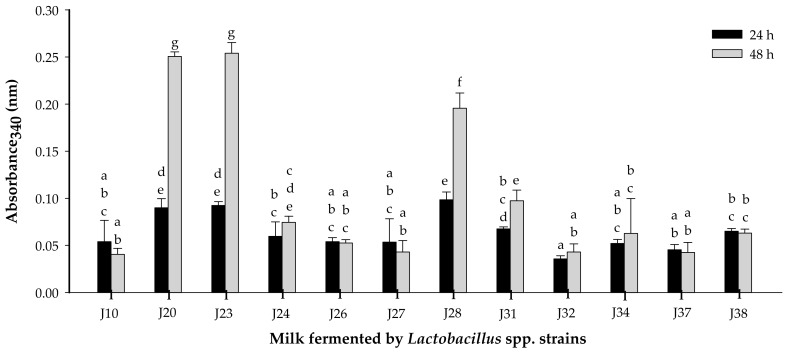
Proteolytic activity (o-phtaldialdehyde method) in milk fermented by different strains of *Lactobacillus* spp. at 24 or 48 h of fermentation (37 °C). Mean ± SD (*n* = 3). Different letters indicate significant differences (*p* < 0.05) among all fermented milks. Strains: J10, J20, J23, J28, J32 and J38 are *Lactobacillus fermentum*; J24, J26, J27, J31, J34 and J37 are *Lactobacillus pentosus*.

*Lactobacillus* strains from two species (six *L. fermentum* and six *L. pentosus*) were evaluated [19]. Proteolytic activity assessed at 24 and 48 h of fermentation showed significant differences (*p* < 0.05), and it was time and strain dependent (Figure 1). Strains of *Lactobacillus fermentum* J20, J23 and J28 presented the highest proteolytic activity (*p* < 0.05). On the other hand, strains J10, J24, J26, J27, J31, J32, J34, J37 and J38 were considered as weakly proteolytic. The high proteolytic activity for species *L. fermentum* may be related to a large number of proteases and peptidases present [24].

It has been reported that metabolites such as peptides, released during fermentation, are important for the bioactive properties attributed to highly proteolytic lactic acid bacteria [8]. In fact, a study of the effect of *L. helveticus* and its nonproteolytic variant reported that mice administrated with milk fermented by the proteolytic variant (wild) of *L. helveticus* enhanced the immunomodulatory effect in comparison to those fed with milk fermented with the nonproteolytic variant [8]. Moreover, in another study the peptidic fractions from *Lactobacillus helveticus-*fermented milk offered protection against *Salmonella* infection, possibly by interfering in the virulence function [9] 

#### 3.1.2. Acidifying Activity

**Figure 2:**
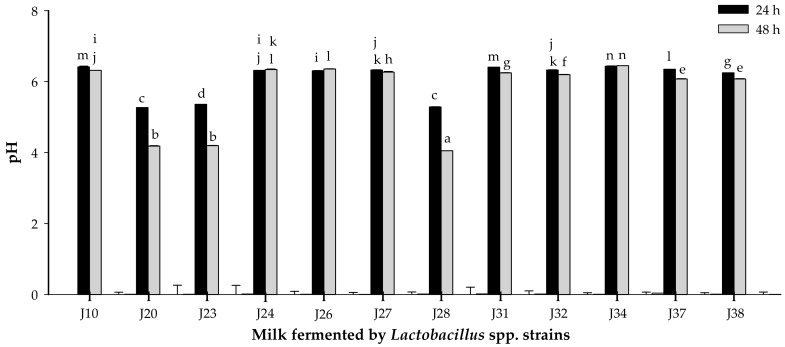
pH of milk fermented by *Lactobacillus* spp. strains at 24 or 48 h of fermentation. Mean ± SD (*n* = 3). Different letters indicate significant differences (*p* < 0.05) among all fermented milks. Strains: J10, J20, J23, J28, J32 and J38 are *Lactobacillus fermentum*; J24, J26, J27, J31, J34 and J37 are *Lactobacillus pentosus*.

**Figure 3:**
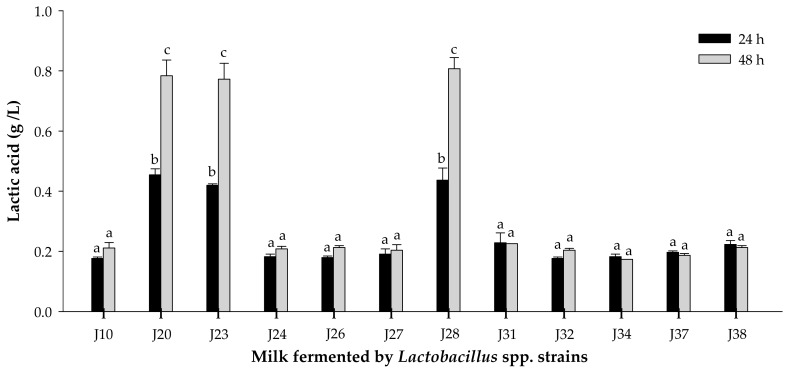
Lactic acid concentration in milk fermented by *Lactobacillus* spp. strains at 24 or 48 h of fermentation. Mean ± SD (*n* = 3). Different letters indicate significant differences (*p* < 0.05) among all fermented milks. Strains: J10, J20, J23, J28, J32 and J38 are *Lactobacillus fermentum*; J24, J26, J27, J31, J34 and J37 are *Lactobacillus pentosus*.

The acidifying activity of *Lactobacillus* strains was evaluated by monitoring pH and titratable acidity at 24 and 48 h of fermentation (Figure 2 and Figure 3). pH significantly decreased (*p* < 0.05) for milk fermented by *Lactobacillus* J20, J23 and J28 after 24 or 48 h. Similarly, titratable acidity was significantly (*p* < 0.05) different for these same strains of *Lactobacillus* J20, J23 and J28 after 24 and 48 h of fermentation. On the other hand, pH and titratable acidity did not significantly (*p* > 0.05) change for the rest of the strains. pH change and lactic acid production were strain dependent and may be explained in terms of differences in metabolic ability and growth requirements. While pH reduction depends on the amount of lactic acid and other organic acids released, which is directly linked to the culture metabolic capacity, titratable acidity depends only on the lactic acid produced [25].

Since *Lactobacillus* J20, J23 and J28 showed the highest proteolytic and acidifying activities in milk, which are desirable technological properties; they were selected for further studies. Efficient proteolytic activity of some *Lactobacillus* sp. has been associated to the production of bioactive peptides with immunomodulatory activity derived from the hydrolysis of casein in milk. Additionally, acidification protects milk against spoilage by microorganisms and proliferation of pathogens, as well as contributes to the flavor and texture of fermented dairy products [25].

Bacterial growth for the selected strains of *L. fermentum* J20, J23 and J28 was monitored, and the point corresponding to the end of the exponential phase that reached 10^9^ CFU/mL at 12 h was chosen as the initial average population for milk inoculum.

### 3.2. Cytokine Analysis in an LPS-Stimulated Murine Model

In this study, the effects of milk fermented by the three different selected strains of *L. fermentum* were evaluated, according to their capacity to modulate the production of IL-6, TNF-α and IL-10 cytokines in a LPS-stimulated murine model.

**Figure 4:**
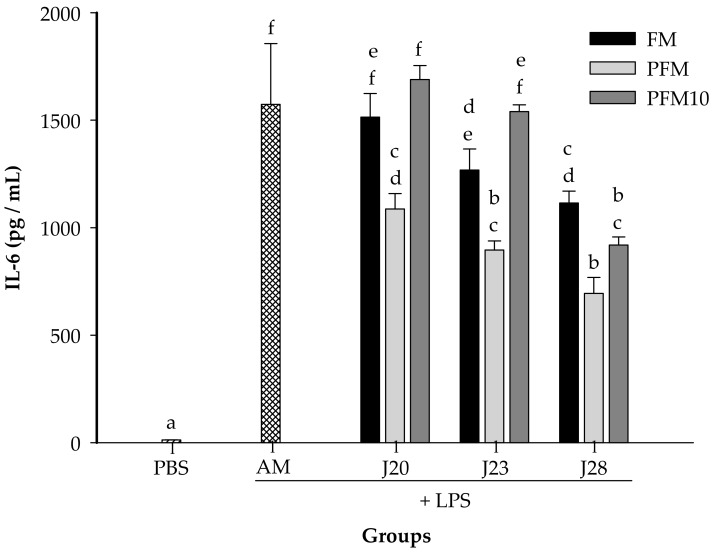
Serum concentrations of IL-6. Wistar rats were fed with fermented milk (FM), pasteurized fermented milk (PFM), fractions <10 kDa of PFM (PFM10) with *Lactobacillus* J20, J23 or J28; after 4 weeks, rats were injected with lipopolysaccharide, and sacrificed after 6 h post-injection. Control groups included: acidified milk (AM) injected with LPS; or phosphate buffer saline (PBS) without LPS. Bars represent means ± SE (*n* = 6). Different letters indicate significant differences (*p* < 0.05) among all groups.

**Figure 5:**
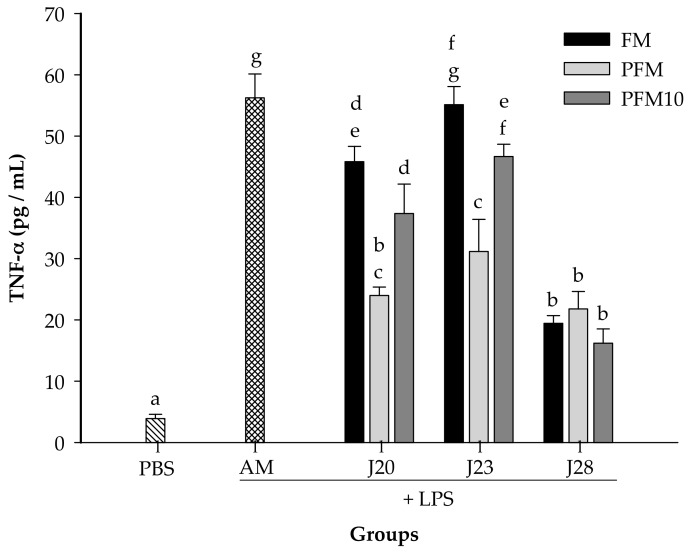
Serum concentrations of TNF-α. Groups were daily administrated for 4 weeks with fermented milk (FM), pasteurized fermented milk (PFM), fractions <10 kDa of PFM (PFM10) with *Lactobacillus* J20, J23 or J28; after 4 weeks, rats were injected with LPS, and sacrificed after 6 h post-injection. Control groups included: acidified milk (AM) injected with LPS; or phosphate buffer saline (PBS) without LPS. Bars represent means ± SE (*n* = 6). Different letters indicate significant differences (*p* < 0.05) among all groups.

**Figure 6:**
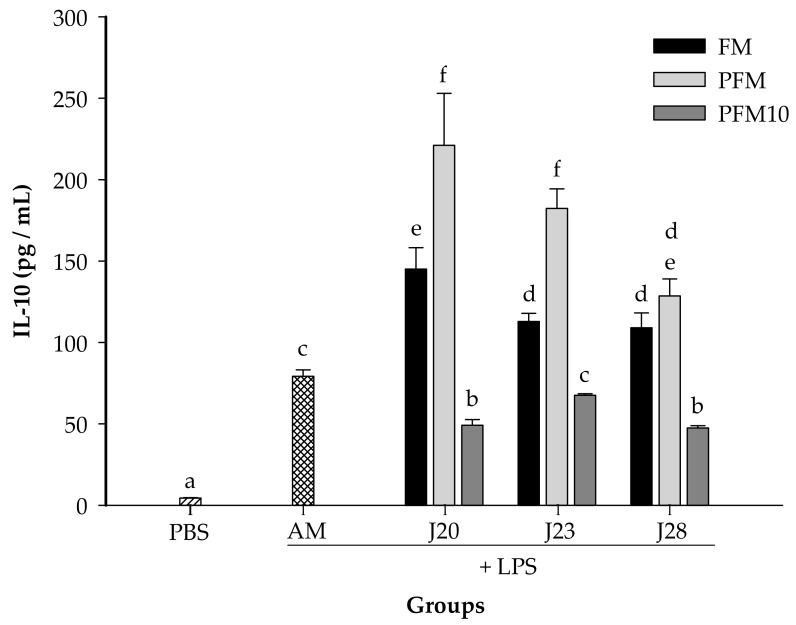
Serum concentration of IL-10. Groups were daily administrated for 4 weeks with fermented milk (FM), pasteurized fermented milk (PFM), fractions <10 kDa of PFM (PFM10) with *Lactobacillus* J20, J23 or J28; after 4 weeks, rats were injected with LPS, and sacrificed after 6 h post-injection. Control groups included: acidified milk (AM) injected with LPS; or phosphate buffer saline (PBS) without LPS. Bars represent means ± SE (*n* = 6). Different letters indicate significant differences (*p* < 0.05) among all groups.

In a preliminary assay, it was found that 7.5 mg/kg of BW of LPS was an adequate dose to induce an increase on cytokine production in serum (data not shown). As expected, for all the cytokines tested, the LPS-stimulated control group (AM) presented significantly higher (*p* < 0.05) levels than the PBS group (without LPS stimulation) (Figure 4, Figure 5 and Figure 6).

The IL-6 concentrations were determined in animals treated with LPS (Figure 4). Groups fed with FM/J23 or FM/J28 presented significantly lower (*p* < 0.05) IL-6 in serum, in contrast to the control group. However, FM/J20 did not show significant differences (*p* > 0.05) in IL-6 compared to the control group (AM). Furthermore, IL-6 concentrations in animals treated with PFM/J20, PFM/J23 or PFM/J28 were significantly (*p* < 0.05) lower than the control group (AM). Also, results showed that groups treated with PFM10/J28 significantly decreased (*p* < 0.05) concentrations of IL-6 in comparison to the control group. Nevertheless, PFM10/J20 and PFM/J23 were not significantly different (*p* > 0.05) from the control group.

In the case of TNF-α concentrations (Figure 5), animals treated with FM/J20 or FM/J28 showed a reduction (*p* < 0.05) of TNF-α compared to the control group. Nevertheless, FM/J23 was not significantly (*p* > 0.05) different from the control group. Also, all PFM/J20, PFM/J23 or PFM/J28 treatments significantly reduced (*p* < 0.05) TNF-α concentrations when compared to the control group. Moreover, results showed that groups treated with PFM10/J20, PFM/J23, and PFM/J28 significantly decreased (*p* < 0.05) concentrations of pro-inflammatory TNF-α cytokines, in comparison to the control group (AM) (Figure 5). It is noteworthy that FM/J28 was able to reduce TNF-α in all the treatments, whether in fermented milk, pasteurized fermented milk or its fractions.

Furthermore, the IL-10 concentrations were determined post-LPS treatments (Figure 6). Animals treated with FM/J20, FM/J23 or FM/J28 presented IL-10 concentrations that were significantly (*p* < 0.05) higher than the control group. Moreover, serum concentrations of this cytokine from PFM with J20, J23 or J28 groups were also significantly (*p* < 0.05) higher than those in the control group. Nevertheless, the IL-10 concentration for all administered groups with PFM10 did not increase in comparison to the control group (Figure 6). This may be due to the fact that the peptidic nitrogen content in PFM10/J28 was significantly (*p* < 0.05) lower than that in PFM (0.20 vs. 0.30%).

### 3.3. Identification of Peptides in Milk Fermented by Lactobacillus Fermentum J28 with Potential Regulatory Effect on Cytokine Production

To the best of our knowledge, this is the first study that reports the anti-inflammatory effect of fermented milk with wild lactic acid bacteria different from *L. helveticus.* Indeed, the anti-inflammatory effect of FM with *L. helveticus* was reported in a murine model with LPS-induced inflammation and demonstrated that after three weeks of treatments, there was an inhibition of the production of the pro-inflammatory cytokine TNF-α, and an enhancement of the production of anti-inflammatory cytokine IL-10 [26].

The beneficial effects of fermented milk products may not only be attributed to bacteria themselves, but also to the metabolites produced during the fermentation [7]. The most important metabolites in fermented milks are peptides not present prior to fermentation; these bioactive peptides are potential modulators of various regulatory processes in the body [27]. In the present study, PFM treatment containing inactive bacteria presented a cytokine regulatory effect; therefore, this effect may not be attributable to live bacteria. It has been reported that nonviable bacteria may also exert beneficial effects [28]. In this sense, studies have reported that heat-treated whole nonviable bacterial cells, such as *L. casei Shirota* [29], *L. acidophilus* A2, *L. gasseri* A5, and *L. salivarius* A6 [30] presented anti-inflammatory effect. Thus, the anti-inflammatory effect of bacterial components present in PFM cannot be discarded.

Moreover, the regulatory effect of fermented milk was enhanced with heat treatment, which might be associated to structural protein changes. It has been reported that heat treatment changes the structural conformation of proteins and peptides in milk, promoting the formation of a large variety of bioactive peptides through digestion, since different protein bonds will be available for enzymes in the gastrointestinal tract [31]. Thus, peptides and proteins present in PFM may be more readily available for digestion in order to exert the regulatory effect. In fact, pro-inflammatory cytokine concentrations in PFM tended to be lower than in FM (Figure 4 and Figure 5).

It is important to note that milk fermented by J28 (FM or PFM) was the most potent regulator for pro-inflammatory cytokines (IL-6 and TNF-α), while groups treated with FM/J20 or PFM/20 and PFM/23 were more potent inducers for the anti-inflammatory cytokine IL-10. Furthermore, it is worthwhile to highlight that although the proteolytic activity of J28 was significantly (*p* < 0.05) lower than that for J20 or J23 (Figure 1), treatments with J28, seem to have the greatest capacity to regulate pro-inflammatory cytokine production. Thus, it appears that what it is most important is the chemical structure of bioactive peptides.

It has been reported that peptides with the capacity to regulate the production of cytokines or the proliferation of immune cells consist of 2 to 32 AA, which may be contained in the fraction with a molecular weight of <10 kDa [32,33]. Hence, peptides present in PFM10 with J28 were identified.

**Figure 7:**
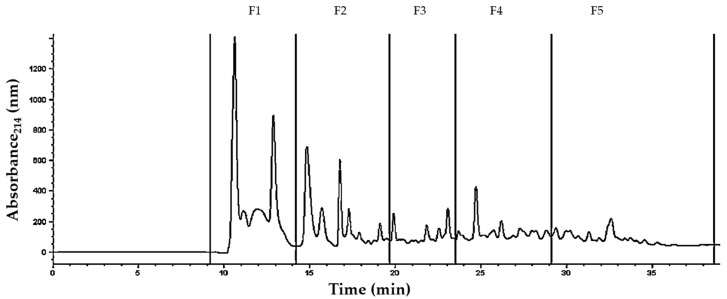
Peptide profiles in the water soluble fraction (<10 kDa) from pasteurized fermented milk (PFM10) with *Lactobacillus fermentum* J28, obtained by reversed-phase High Performance Liquid Chromatography at 214 nm. F1 to F5 correspond to collected fractions.

**Figure 8:**
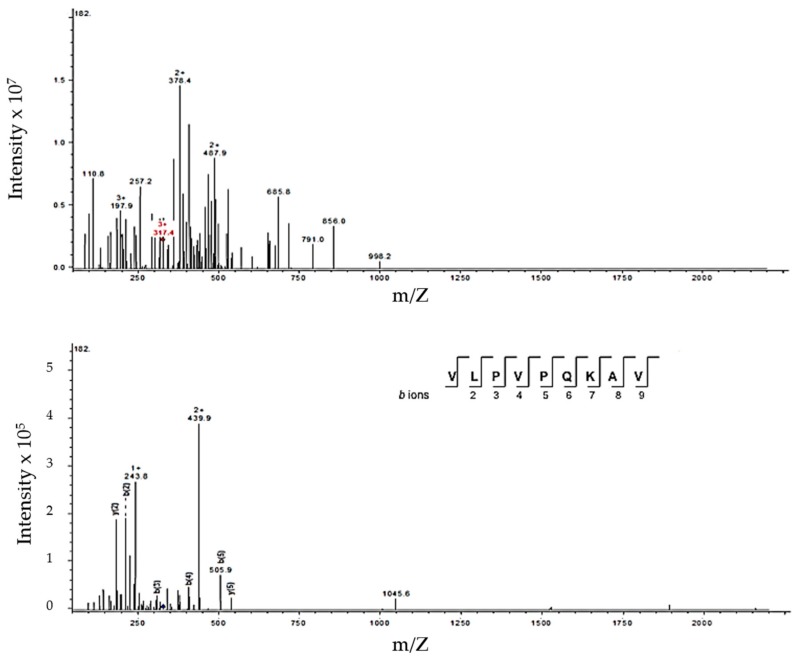
Typical mass spectrum corresponding to peptide sequence (VLPVPQKAV) collected from F4 obtained from milk fermented by *Lactobacillus fermentum* J28. (**A**) Triple-charged ion 317.4 *m*/*z*; (**B**) tandem mass spectrometry (MS/MS) spectrum for the specified ion in (**A**). After interpretation and comparison in the database, the fragment AA sequence matched β-CN (f185-193).

A typical peptide profile produced by J28 showing five collected fractions (F1 to F5) is depicted in Figure 7. After fraction collection, peptides were identified by mass spectrometry. A typical mass spectrum of one of the peptides derived from β-casein is depicted in Figure 8. The proteolysis process gave rise to medium-sized peptides, ranging in length from 7 to 32 amino acids and a molecular weight mostly <3 kDa (Table 1).

Proteases and peptidases in J28 targeted mainly whey proteins, since twelve peptide sequences were derived from whey and only three from caseins. Peptides listed in Table 1 showed the action of mainly serine proteases, specifically trypsin, which cleaves the peptide chains mainly at the carboxyl side of the amino acids lysine (K) or arginine (R) [34].

A key point in the cytokine regulatory effect of bioactive peptides is the type of amino acid in the N-terminal and C-terminal positions [35]. Evidence suggests that the amino acid R in the extreme (N-terminal or C-terminal) of bioactive peptides is the dominant entity recognized by receptors on macrophages and lymphocytes, which may enhance their maturation and proliferation [36,37]. In this study, only one sequence with R (RYPSYGLN) derived from casein was present in milk fermented by J28. Portions of this sequence (YPSYGL and YPSYG) were reported to have angiotensin converting enzyme inhibitory activity (ACEI), in milk fermented by *Lactococcus lactis* NRRL B-50571 [38]. Also, sequence RYPSYG was reported to have ACEI in bovine casein hydrolysate prepared by neutral protease [39].

Similarly, a peptide (VLPVPQKAV) from β-casein was identified (Figure 8). It was reported that a portion of this peptide VLPVPQ presented ACEI activity [40]. Also, another peptide (VLPVPQK) derived from this same sequence inhibited lipoxygenase (enzyme associated to inflammatory process) activity in vitro. Thus, this peptide was linked to modulating inflammation [41]. Furthermore, the peptide VLPVPQK has also been reported to have anti-oxidative potential effect [42]. Additionally, it was reported that this peptide may be absorbed intact with PepT1-like transporters in a human intestinal cell model (Caco-2) [43]. Milk derived peptides have different biological effects, such as antihypertensive (ACEI), antioxidant and immunomodulatory [42,44]. Thus, one particular peptide sequence may have multiple bioactivities. These peptides, are inactive in the milk protein, and may be released and activated through milk fermentation by microorganisms and gastrointestinal digestion [45].

Likewise, the antihypertensive peptide VY [45] and the hypotriglyceridemic peptide VTL, which may be liberated during gastrointestinal digestion [46], are contained in the whey protein derived peptides, such as LDAQSAPLRVYVEELKPT and ADAVTLDGGMVFEAGRDPYKLRPVAAEIYGTK, respectively (Table 1). Additionally, the dipeptide YG contained in this sequence was reported to enhance the proliferation of peripheral blood lymphocytes and be used as an immunomodulatory peptide [47].

When LPS is released into the bloodstream causes inflammation via activation of monocytes and endothelial cells. It can lead to septic shock and even death. One strategy to prevent endotoxic shock is to neutralize the negatively charged phosphoryl groups present in lipid A, the most conserved part and major mediator of LPS activity bear by positively charged LPS-binding molecules, such as proteins or peptides [48]. In this sense, some examples of dairy peptides with proven LPS-binding ability that can reduce LPS activity have been reported [49,50]. Nevertheless, further studies are needed to evaluate the possible LPS-binding activity of peptides from milk fermented by J28.

The literature has reported the immunomodulatory effect of peptides mainly derived from caseins, and just few were related to the anti-inflammatory process [35]. Thus, these results open the possibility for finding new peptide sequences with anti-inflammatory activity.

In conclusion, FM by specific strains of *L. fermentum* are able to modulate the balance of LPS-induced pro- and anti-inflammatory serum cytokines. The cytokine regulatory effect was possibly due to components released in fermented milk, since pasteurized fermented milk and fractions <10 kDa also showed the effect. Thus, cytokine regulation may be associated to peptides present in fermented milk, nevertheless the effect of other milk components cannot be discarded. Furthermore, more studies are needed in order to elucidate the components responsible for the observed effect. Thus, fermented milk with these specific strains of *L. fermentum* show potential for the development of novel functional foods for the attenuation of systemic inflammatory disorders.

## Figures and Tables

**Table 1:** Identification of peptides in the water soluble fraction (<10 kDa) obtained from milk fermented by *Lactobacillus fermentum* J28.

Sample *^a^*	Experimental Mass	Theoretical Mass	Molecular Ion (*m*/*z*) Selected for MS/MS *^b^*(Charge)	Protein Fragment	Sequence
F1	1622.75	1623.26	541.1 (+3)	α-La (f100–113)	LDDDLTDDIMCVKK
	2028.52	2028.09	2029.5 (+1)	β-Lg (f48–65)	LDAQSAPLRVYVEELKPT
F2	1877.74	1878.87	1878.7 (+1)	α_S1_-CN (50–66)	EKVNELSKDIGSESTED
	708.21	708.34	237.1 (+3)	α-La (f33–39)	DLKGYGG
F3	1029.86	1030.65	344.3 (+3)	β-Lg (f91–99)	KTKIPAVFK
	729.22	729.43	244.1 (+3)	Lactotransferrin (f67–73)	AIAEKKA
	3409.04	3409.73	1705.5 (+2)	Lactotransferrin (f73–104)	ADAVTLDGGMVFEAGRDPYKLRPVAAEIYGTK
	2036.38	2036.87	2037.4 (+1)	Lactotransferrin (f564–581)	NDTVWENTNGESTADWAK
	810.65	811.40	407.3 (+2)	Serotransferrin (f668–674)	AKKTYDS
	831.44	832.42	832.4 (+1)	Serotransferrin (f683–689)	AMTNLRQ
F4	967.94	968.47	484.9 (+2)	κ-CN (f55–62)	RYPSYGLN
	949.23	949.59	317.4 (+3)	β-CN (185–193)	VLPVPQKAV
	2236.12	2237.17	2237.1 (+1)	Lactotransferrin (f628–647)	QVLLHQQALFGKNGKNCPDK
F5	916.00	916.45	459.0 (+2)	β-Lg (f117–123)	KYLLFCM
	1133.62	1133.50	284.4 (+4)	Lactotransferrin (f523–533)	LCAGDDQGLDK

*^a^* F1 to F5 are collected fractions. *^b^* MS/MS: tandem mass spectrometry. Abbreviations used in table: α-La, alpha-lactalbumin; β-Lg, beta-lactoglobulin; α_S1_-CN, alpha-S1-casein; κ-CN, kappa-casein; β-CN, beta-casein.

## References

[1] Shiby V.K., Mishra H.N. (2013). Fermented Milks and Milk Products as Functional Foods—A Review. Crit. Rev. Food Sci. Nutr..

[2] Chiu Y.H., Lin S.L., Ou C.C., Lu Y.C., Huang H.Y., Lin M.Y. (2013). Antiinflammatory effect of lactobacilli bacteria on HepG2 cells is through cross-regulation of TLR4 and NOD2 signalling. J. Funct. Foods.

[3] Juarez G.E., Villena J., Salva S., Font de Valdez G., Rodriguez A.V. (2013). *Lactobacillus reuteri* CRL1101 beneficially modulate lipopolysaccharide-mediated inflammatory response in a mouse model of endotoxic shock. J. Funct. Foods.

[4] Maldonado Galdeano C., Novotny Núñez I., de Moreno de LeBlanc A., Carmuega E., Weill R., Perdigón G. (2011). Impact of a probiotic fermented milk in the gut ecosystem and in the systemic immunity using a non-severe protein-energy-malnutrition model in mice. BMC Gastroenterol..

[5] Agyei D., Ongkudon C.M., Wei C.Y., Chan A.S., Danquah M.K. (2016). Bioprocess challenges to the isolation and purification of bioactive peptides. Food Bioprod. Process..

[6] LeBlanc J., Fliss I., Matar C. (2004). Induction of a humoral immune response following an *Escherichia coli* O157:H7 infection with an immunomodulatory peptidic fraction derived from *Lactobacillus helveticus*-fermented milk. Clin. Diagn. Lab. Immunol..

[7] Vinderola G., Matar C., Palacios J., Perdigón G. (2007). Mucosal immunomodulation by the non-bacterial fraction of milk fermented by *Lactobacillus helveticus* R389. Int. J. Food Microbiol..

[8] Matar C., Valdez J.C., Medina M., Rachid M., Perdigon G. (2001). Immunomodulating effects of milks fermented by *Lactobacillus helveticus* and its nonproteolytic variant. J. Dairy Res..

[9] Tellez A., Corredig M., Turner P., Morales R., Griffiths M.W. (2011). A peptidic fraction from milk fermented with *L. helveticus* protects mice against *Salmonella* infection. Int. Dairy J..

[10] Sharma R., Kapila R., Kapasiya M., Saliganti V., Dass G., Kapila S. (2014). Dietary supplementation of milk fermented with probiotic *Lactobacillus fermentum* enhances systemic immune response and antioxidant capacity in aging mice. Nutr. Res..

[11] Deng B., Wu J., Li X., Men X., Xu Z. (2017). Probiotics and probiotic metabolic product improved intestinal function and ameliorated lps-induced injury in rats. Curr. Microbiol..

[12] Koscik R.J.E., Reid G., Kim S.O., Li W., Challis J.R.G., Bocking A.D. (2017). Effect of *Lactobacillus rhamnosus* GR-1 Supernatant on cytokine and chemokine output from human amnion cells treated with lipoteichoic acid and lipopolysaccharide. Reprod. Sci..

[13] Shigemori S., Namai F., Yamamoto Y., Nigar S., Sato T., Ogita T., Shimosato T. (2017). Genetically modified *Lactococcus lactis* producing a green fluorescent protein–bovine lactoferrin fusion protein suppresses proinflammatory cytokine expression in lipopolysaccharide-stimulated RAW 264.7 cells. J. Dairy Sci..

[14] Dinarello C.A. (2000). Proinflammatory cytokines. Chest.

[15] Miettinen M., Vuopio-Varkila J., Varkila K. (1996). Production of human tumor necrosis factor alpha, interleukin-6, and interleukin-10 is induced by lactic acid bacteria. Infect. Immun..

[16] Gabay C. (2006). Interleukin-6 and chronic inflammation. Arthritis Res. Ther..

[17] Louis H., LeMoine O., Peny M.O., Quertinmont E., Fokan D., Goldman M., Devière J. (1997). Production and role of interleukin-10 in concanavalin A induced hepatitis in mice. Hepatology.

[18] Sang H., Wallis G.L., Stewart C.A., Kotake Y. (1999). Expression of cytokines and activation of transcription factors in lipopolysaccharide-administered rats and their inhibition by phenyl N-tert-butylnitrone (PBN). Arch. Biochem. Biophys..

[19] Heredia C.P.Y., Méndez-Romero J.I., Hernández-Mendoza A., Acedo-Félix E., González-Córdova A.F., Vallejo-Cordoba B. (2015). Antimicrobial activity and partial characterization of bacteriocin-like inhibitory substances produced by *Lactobacillus* spp. isolated from artisanal Mexican cheese. J. Dairy Sci..

[20] Smith P.K., Krohn R.I., Hermanson G.T., Mallia A.K., Gartner F.H., Provenzano M.D., Fujimoto E.K., Goeke N.M., Olson B.J., Klenk D.C. (1987). Measurement of protein using bicinchoninic acid. Anal. Biochem..

[21] AOAC (2000). Official Methods of Analysis of AOAC.

[22] National Research Council (NRC) (2011). Guide for the Care and Use of Laboratory Animals.

[23] Perkins D., Pappin D.J., Creasy D.M., Cottrell J.S. (1999). Probability-based protein identification by searching sequence databases using mass spectrometry data. Electrophoresis.

[24] Shihata A., Shah N.P. (2000). Proteolytic profiles of yogurt and probiotic bacteria. Int. Dairy J..

[25] Widyastuti Y., Rohmatussolihat, Febrisiantosa A. (2014). The role of lactic acid bacteria in milk fermentation. Food Nutr. Sci..

[26] Zhou J., Ma L., Xu H., Gao Y., Jin Y., Zhao L., David X.A.L., Zhan D., Zhang S. (2014). Immunomodulating effects of casein-derived peptides QEPVL and QEPV on lymphocytes in vitro and in vivo. Food Funct..

[27] Meisel H., Bockelmann W. (1999). Bioactive peptides encrypted in milk proteins: Proteolytic activation and thropho-functional properties. Antonie Leeuwenhoek.

[28] Taverniti V., Guglielmetti S. (2011). The immunomodulatory properties of probiotics microorganisms beyond their viability (ghost probiotics: Proposal of paraprobiotic concept). Genes Nutr..

[29] Cross M.L., Ganner A., Teilab D., Fray L.M. (2004). Patterns of cytokine induction by gram-positive and gram-negative probiotic bacteria. FEMS Immunol. Med. Microbiol..

[30] Chuang L., Wu K.G., Pai C., Hsieh P.S., Tsai J.J., Yen J.H., Lin M.Y. (2007). Heat-killed cells of lactobacilli skew the immune response toward T helper polarization in mouse splenocytes and dentritic cell-treated T cells. J. Agric. Food Chem..

[31] Sánchez-Rivera L., Ménard O., Recio I., Dupont D. (2015). Peptide mapping during dynamic gastric digestion of heated and unheated skimmed milk powder. Food Res. Int..

[32] Gill H.S., Doull F., Rutherfurd K.J., Cross M.L. (2000). Immunoregulatory peptides in bovine milk. Br. J. Nutr..

[33] Requena P., González R., López-Posadas R., Abadía-Molina A., Suárez M.D., Zarzuelo A., de Medina F.S., Martínez-Augustin O. (2010). The intestinal antiinflammatory agent glycomacropeptide has immunomodulatory actions on rat splenocytes. Biochem. Pharmacol..

[34] Rodriguez J., Gupta N., Smith R.D., Pevzner P.A. (2008). Does trypsin cut before proline?. J. Proteome Res..

[35] Reyes-Díaz A., González-Córdova A.F., Hernández-Mendoza A., Reyes-Díaz R., Vallejo-Cordoba B. (2018). Immunomodulation by hydrolysates and peptides derived from milk proteins. Int. J. Dairy Technol..

[36] Meisel H., FitzGerald R.J. (2003). Biofunctional peptides from milk proteins: Mineral binding and cytomodulatory effects. Curr. Pharm. Des..

[37] Haque E., Chand R. (2008). Antihypertensive and antimicrobial bioactive peptides from milk proteins. Eur. Food Res. Technol..

[38] Rodríguez-Figueroa J.C., González-Córdova A.F., Torres-Yanez M.J., Garcia H.S., Vallejo-Cordoba B. (2010). Novel angiotensin I-converting enzyme inhibitory peptides produced in fermented milk by specific wild *Lactococcus lactis* strains. J. Dairy Sci..

[39] Jiang Z., Tian B., Brodkorb A., Huo G. (2010). Production, analysis and in vivo evaluation of novel angiotensin-I-converting enzyme inhibitory peptides from bovine casein. Food Chem..

[40] Hernández-Ledesma B., Amigo L., Ramos M., Recio I. (2004). Angiotensin converting enzyme inhibitory activity in commercial fermented products. Formation of peptides under simulated gastrointestinal digestion. J. Agric. Food Chem..

[41] Rival S.G., Boeriu C.G., Wichers H.J. (2001). Caseins and casein hydrolysates. 2. Antioxidative properties and relevance to lipoxygenase inhibition. J. Agric. Food Chem..

[42] Shanmugam V.P., Kapila S., Kemgang T.S., Kapila R. (2015). Antioxidative peptide derived from enzymatic digestion of buffalo casein. Int. Dairy J..

[43] Vij R., Reddi S., Kapila S., Kapila R. (2016). Transepithelial transport of milk derived bioactive peptide VLPVPQK. Food Chem..

[44] Qian B., Xing M., Cui L., Deng Y., Xu Y., Huang M., Zhang S. (2011). Antioxidant, antihypertensive, and immunomodulatory activities of peptide fractions from fermented skim milk with *Lactobacillus delbrueckii* ssp. *bulgaricus* LB340. J. Dairy Res..

[45] Matsui T., Tamaya K., Seki E., Osajima K., Matsumo K., Kawasaki T. (2002). Absorption of Val-Tyr with in vitro angiotensin i-converting enzyme inhibitory activity into the circulating blood system of mild hypertensive subjects. Biol. Pharm. Bull..

[46] Kagawa K., Matsutaka H., Fukuhama C., Watanabe Y., Fujino H. (1996). Globin digest, acidic protease hydrolysate, inhibits dietary hypertriglyceridemia and Val-Val-Tyr-Pro, one of its constituents, possesses most superior effect. Life Sci..

[47] Kayser H., Meisel H. (1996). Stimulation of human peripheral blood lymphocytes by bioactive peptides derived from bovine milk proteins. FEBS Lett..

[48] Van Amersfoort E.S., Van Berkel T.J.C., Kuiper J. (2003). Receptors, mediators, and mechanisms involved in bacterial sepsis and septic shock. Clin. Microbiol. Rev..

[49] Cheng X., Gao D., Chen B., Mao X. (2015). Endotoxin-binding peptides derived from casein glycomacropeptide inhibit lipopolysaccharide-stimulated inflammatory responses via blockade of NF-κβ activation in macrophages. Nutrients.

[50] Iskandar M.M., Dauletbaev N., Kubow S., Mawji N., Lands L.C. (2013). Whey protein hydrolysates decrease IL-8 secretion in lipopolysaccharide (LPS)-stimulated respiratory epithelial cells by affecting LPS binding to Toll-like receptor 4. Br. J. Nutr..

